# Rho-Associated Protein Kinases Play an Important Role in the Differentiation of Rat Adipose-Derived Stromal Cells into Cardiomyocytes In Vitro

**DOI:** 10.1371/journal.pone.0115191

**Published:** 2014-12-18

**Authors:** Lili Zhao, Gongshe Yang, Xin Zhao

**Affiliations:** 1 College of Animal Science and Technology, Northwest A&F University, Yangling, Shaanxi, People's Republic of China; 2 Department of Animal Science, McGill University, Ste. Anne de Bellevue, Quebec, Canada; University of Tampere, Finland

## Abstract

Adipose-derived stromal cells (ADSCs) represent a readily available abundant supply of mesenchymal stem cells and have the ability to differentiate into cardiomyocytes in mice and human, making ADSCs a promising source of cardiomyocytes for transplantation. However, there has been no report of differentiation of rat ADSCs into cardiomyocytes. In addition, signaling pathways in the differentiation process from ADSCs to cardiomyocytes are unknown. In this study, we first demonstrated that rat ADSCs spontaneously differentiated into cardiomyocytes in vitro, when cultured on a complete medium formulation MethoCult GF M3534. These differentiated cells possessed cardiomyocyte phenotype and expressed cardiac markers. Moreover, these cells showed open excitation-contracting coupling and Ca^2+^ transient and contracted spontaneously. The role of Rho-associated protein kinases (ROCKs) in the differentiation process was then studied by using ROCK-specific inhibitor Y-27632 and ROCK siRNAs. These agents changed the arrangement of cytoskeleton and diminished appearance of cardiomyocyte phenotype, accompanied by inhibition of c-Jun N-terminal kinase (JNK) phosphorylation and promotion of Akt phosphorylation. Collectively, this is the first study to demonstrate that rat ADSCs could spontaneously differentiate into cardiomyocytes in vitro and ROCKs play an important role in the differentiation of ADSCs into beating cardiomyocytes in conjunction of the PI3K/Akt pathway and the JNK pathway.

## Introduction

Myocardial infarction (MI) afflicts millions of people each year. It causes a significant amount of deaths, especially in developed countries and increasingly more in developing countries. In many of the survivors, MI leads to marked reduction of cardiomyocytes and impaired cardiac pump functions, finally progressing to congestive heart failure. Cell transplantation and gene transfer are two of the foremost therapies with a potential for regenerating damaged cardiomyocytes and enabling revascularization. Among potential cell sources, adipose tissue-derived stromal cells (ADSCs) represent an abundant, practical and appealing source of donor tissue for autologous cell replacement for ischemic heart diseases [1]. A hallmark of the ADSCs is their multi-potency. Cultured ADSCs can be differentiated into adipogenic, osteogenic, chondrogenic, and myogenic cells under certain conditions [2], [3]. Hence, adipose tissue is an attractive cell source for stem cell-based treatment of injured myocardium because it is relatively easy to harvest from patients by a simple, minimally invasive method, available in sufficient quantities and easily cultured.

A putative stem cell population was identified in adipose tissue in 2002 [4]. This discovery opened the door for using adipose tissues as a potential source for obtaining different types of cells. The differentiation of cardiomyocytes from ADSCs was first reported in the rabbit in 2003 by Rangappa et al. [5]. They treated the cultured mesenchymal cells with the DNA demethylation agent 5-azacytidine and confirmed that adult mesenchymal stem cells isolated from fatty tissue could be chemically transformed into cardiomyocytes in vitro. Using a semisolid methylcellulose medium (MethoCult GF M3534), Planat-Bénard et al. [6] obtained beating cardiomyocytes from differentiation of mouse ADSCs. Human ADSCs have also been shown to differentiate into beating cardiomyocytes when co-cultured with beating cardiomyocytes [7]. Nevertheless, there has been no report of differentiation of ADSCs into beating cardiomyocytes in rats. We have previously reported that mouse ADSCs could spontaneously develop into beating cardiomyocytes in DMEM+20%NBS [8]. However, the same culture condition did not induce rat ADSCs into beating cardiomyocytes (data not shown). In addition, dedifferentiated mouse fat (DFAT) cells can differentiate into spontaneously beating cardiomyocytes [9], while rat DFAT cells could not [10]. Therefore, it appears that rat ADSCs are less likely to spontaneously differentiate into beating cardiomyocytes than mouse ADSCs. Nevertheless, the rat offers many advantages over the mouse and other organisms as a model of human disease, especially for cardiovascular diseases [11]. In cardiovascular research, the rat has been the main model of choice for decades. Experimental procedures were developed to generate cardiovascular disease states in this species, such as systemic and pulmonary hypertension, cardiac hypertrophy and failure, myocardial infarction, and stroke. Furthermore, rats have been bred, which spontaneously develop such diseases [12]. While rats share many of the benefits of mice (such as low cost and ease of handling), their larger size greatly facilitates surgical and postsurgical procedures [13]. The physiology is also easier to be monitored in the rat than in the mouse. Moreover, inmany cases, the physiology is more like the corresponding human condition. In addition, the rat has been used as a model animal for cardiac cell transplantation. For example, Yamada et al. [14] isolated CD29+ cells from rat brown adipose tissue and showed that these progenitor cells could differentiate into cardiomyocytes in vitro and in vivo, based on immunohistochemical analyses of cardiomyocytes. The same research group further demonstrated that CD133+, but not c-Kit- or Sca-1-, cells in brown adipose tissue differentiated into cardiomyocytes [15]. However, they did not examine whether these claimed cardiomyocytes could contract or not. Moreover, brown adipose tissue does not exist in great abundance in the adult human. Therefore, brown adipose tissue itself may not have a clinical application for myocardial disease in adult patients. Finally, Danoviz et al. [16] observed that intramyocardial injection of ADSCs 24 hours post-MI significantly preserved left ventricular functions, as assessed by direct hemodynamic evaluation in a rat MI model. However, it is not clear that such an improvement is due to differentiation of injected ADSCs into functional cardiomyocytes or due to factors secreted by ADSCs, such as vascular endothelial growth factor (VEGF) and hepatocyte growth factor (HGF) [1]. It appears that there is no evidence to confirm that rat ADSCs can differentiate into functional cardiomyocytes. This hinders the use of rats as a model of human cardiovascular diseases. Thus, obtaining beating rat cardiomyocytes in vitro could expedite the research of cardiovascular diseases in a rat model.

One of the changes during differentiation of ADSCs into cardiomyocytes is changing of fibroblast-like cells into spherical, tubular-shape cells or myotube-like structure [6]. It is well known that actin cytoskeleton regulates cell morphology. Accumulating experimental findings have suggested the importance of actin cytoskeleton and cell shape in the regulation of cell differentiation and functions [17], [18]. Cell shape is regulated primarily by reorganization of actin cytoskeleton under control of Rho-associated kinases (ROCKs). ROCKs mediate Rho GTPases' signaling and reorganize actin cytoskeleton through phosphorylation of several substrates that contribute to assembly of actin filaments and contractility. ROCKs inhibitor Y-27632 could promote myogenic differentiation in C2C12 myoblasts [19]. By contrast, ROCKs maybe prevent terminal myocardial differentiation during embryonic development [20]. Thus, we have been interested in role of ROCKs in the process of differentiation of ADSCs into cardiomyocytes.

In the present study, we first evaluated whether rat ADSCs could differentiate into functional cardiomyocytes in vitro. Next, we determined the potential role of ROCKs in the differentiation process, by adoption of ROCK-specific inhibitor Y-27632 and ROCK small-interfering RNAs (siRNAs).

## Materials and Methods

### Cell culture and cardiomyocyte differentiation

Cells from the stromal vascular fraction (SVF) were isolated according to Planat-Bénard et al. [6], with slight modifications. Briefly, subcutaneous adipose tissue was obtained from 18-day-old male Sprague Dawley rats (the Fourth Military Medical University, XiAn, China). Animals were sacrificed by cervical dislocation under ethyl ether anesthesia. Subcutaneous adipose tissue was isolated, minced and digested with 0.1% collagenase type I (1 mg/mL collagenase type I (Invitrogen, Carlsbad, CA, USA) and 20 mg/mL BSA) for 40 to 50 min at 37°C on a shaking water bath kettle. After neutralization with DMEM containing 10% of fetal bovine serum, the digested tissue was first centrifuged at 1500 rpm for 7 min and the top lay of fat was removed. The remaining digested tissue was passed through 30-µm nylon mesh, and then centrifuged at 1500 rpm for another 7 min. The supernatant was discarded and the pelleted cells were washed once in PBS and centrifuged. Isolated SVF cells were suspended in PBS and counted with a hemocytometer. Isolated cells were plated (Day 0) at 7.5×10^3^ cells/cm^2^ in 1.5 mL of MethoCult GF M3534 (Stemcell Technologies, Vancouver, BC, Canada) supplemented with 50 U/ml penicillin and 50 U/ml streptomycin. The cells were cultured at 37°C in a humidified atmosphere containing 5% CO_2_, and observed every 1–2 days under an inverted phase-contrast microscope.

In order to determine the role of ROCKs in the differentiation process, ROCK inhibitor (Y-27632, 10 mmol/L; Sigma-Aldrich, Oakville, ON, Canada), a selective ROCK inhibitor, was added to the medium in the treated group. In addition, ROCKI and ROCKII siRNAs were also used in the experiment. The siRNAs and a scrambled control (All stars Negative control siRNA, SCR siRNA) were from Biomics Biotechnologies Co. Ltd. (Nantong, Jiangsu, China). For RNA interference assays, SVF were seeded in six-well plates at 7.5×10^3^ cells/cm^2^ in 1.5 mL of MethoCult GF M3534. The cells were cultured at 37°C in a humidified atmosphere containing 5% CO_2_. Cells were transfected at 80% confluence in a serum-containing medium (DMEM with 10% fetal bovine serum) without antibiotics. Transfections were carried out using Lipofectamine 2000 Transfection Reagents according to the manufacturer's protocol (Invitrogen). Six hours post-transfection, the medium was replaced with MethoCult GF M3534, containing 50 U/ml penicillin and 50 U/ml streptomycin. In preliminary experiments, three independent ROCKI siRNAs and three independent ROCKII siRNAs were investigated for their efficiencies to inhibit ROCKI and ROCKII, respectively. While all siRNA inhibited respective ROCK, siROCKI-3 (87.2% decrease in mRNA levels of ROCKI) and siROCKII-1(91.7% decreasein mRNA levels of ROCKII) were used in later experiments due to their highest efficiencies. The sequences for siROCKI-3 and siROCKII-1are listed in Table 1.

**Table 1:** Primers for reverse transcription PCR and real time PCR and sequences for RNA interference.

Gene	RT-PCR primers (5′-3′)	Product size (bp)
*α-cardiac actin*	F: CTGGATTCTGGCGATGGTGTA	173
	R: CGGACAATTTCACGTTCAGCA	
*α-MHC*	F: GGAAGAGCGAGCGGCGCATCAAGG	304
	R: CTGCTGGACAGGTTATTCCTCA	
*β-MHC*	F: GCCAACACCAACCTGTCCAAGTTC	202
	R: TTCAAAGGCTCCAGGTCTCAGGGC	
*ANP*	F: GCCGGTAGAAGATGAGGTCA	269
	R: GGGCTCCAATCCTGTCAATC	
*cTnT*	F: GGGTACATCCAGAAGGCTCA	374
	R: GTGCCTGGCAAGACCTAGAG	
*GATA4*	F: AGCAAGGACTAGGCACCTCTAGC	412
	R: ATAGCCAGGCTTTGGTACATCGC	
*MEF-2C*	F: CCGATGCAGACGATTCAGTAG	260
	R: GTGTCACACCAGGAGACATAC	
*ROCK I*	F: TGATGGCTATTATGGACGAGA	201
	R: GTAAGGAAGGCACAAATGAGAT	
*ROCK II*	F: GATGGCTGTCGTTGCCTGTG	126
	R: AAGGGTTAGACTGCTCTTTATCTTGTTC	
*β-actin*	F: CCCATCTATGAGGGTTACGC	150
	R: TTTAATGTCACGCACGATTTC	
*ROCKI* siRNA-3	F: GCAAAUCAGUCUUUCCGGAdTdT	
	R: UUCUAUUCGAAUUUGCdTdT	
*ROCKII* siRNA-1	F: GAGCCAAAUUCGAAUAGAAdTdT	
	R: UUCUAUUCGAAUUUGGCUCdTdT	
Negative control	F: UUCUCCGAACGUGUCACGUdTdT	
	R: ACGUGACACGUUCGGAGAAdTdT	

All procedures were approved by the Institutional Animal Care and Use Committee of Northwest A&F University.

### Gene expression analyses

Expression of cardiac genes (*α-cardiac actin, α-MHC, β-MHC, ANP, cTnT, GATA4, and MEF-2C*) was determined by reverse transcription (RT)-PCR. Contracting cells were identified and separated under the microscope. Total RNA was extracted using RNAiso plus reagent (TaKaRa Biotechnology, Dalian, Liaoning, China). Five hundred ng of total RNA was processed into single strand cDNA using reverse transcription kits (Takara) with random primers. The RT-PCR was performed for 28 cycles using the Taq Plus MasterMix (Tiangen Biotech, Beijing, China) in a GeneAmp PCR system. Expression of *ROCKI* and *ROCKII* genes was determined by real time PCR. The real time PCR reactions were performed in triplicates using the SYBR green kit (TaKaRa Biotechnology) with a Bio-rad iQ5 system. The 2^-ΔΔCT^ method was used to analyze the relative expression of each gene [8]. The primers sequences and product size for PCR are listed in Table 1.

### Immunofluorescence

Before the immunofluorescence analysis, contracting cells were identified and marked under the microscope. For immunofluorescence analyses of cardicmarkers cardiac troponinI (cTnI), Nkx2.5, cardiac myosinheavy chain (cMHC), α-actin, non-cardiac muscle proteins (MyoD for skeletal muscle and α-smooth muscle actin(α-SMA) for smooth muscle), beating cells were selected and washed with PBS, fixed with 4% paraformaldehyde, permeabilized with 0.1% Triton X-100, blocked with 10% goat serum and 5% BSA in PBS, and incubated overnight at 4°C with anti-cMHC (1∶100; Beijing Biosynthesis Biotechnology, Beijing, China), anti-cTnI (1∶100; Beijing Biosynthesis Biotechnology), anti-Nkx2.5 (1∶50; Santa Cruz Biotechnology), anti-α-actin (1∶50; Santa Cruz Biotechnology), anti-MyoD (1∶50; Santa Cruz Biotechnology), or anti-α-smooth muscle actin (1∶100; Wuhan Boster Biological Technology). At room temperature, cells were washed three times with PBS, incubated for 60 min with Cy3 or FITC-conjugated secondary antibodies, washed three times with PBS, and stained for 5 min with Hoechst 33342 (0.5 µg/mL, Sigma, St. Louis, MO, USA). Finally, the cells were washed three times with PBS. Images were visualized and obtained by a Nikon TE 2000 microscope. The immune-staining methods were validated by using positive and negative control tissues [8].

For cytoskeleton staining, cells grown in culture dishes were washed with PBS, fixed with 4% paraformaldehyde for 10 min, and then washed three times with PBS containing 0.1% Triton X-100. F-actin was stained with Actin-Tracker Green (phalloidin, Beyotime Institute of Biotechnology, Guangzhou, China).

### Flow cytometry

Cardiac proteins (DHPR, RyR2, cTnI, Connexin45, MLC-2v, cTnT and cMHC) were also detected by flow cytometry. The cells after 3 days of culture (no differentiation at this time) or after appearance of first beating were digested with 0.25% trypsin, fixed with 4% paraformaldehyde, permeabilized with 0.1% Triton X-100, and incubated for 1 h at 4°C with anti-DHPR (1∶50), anti-RyR2 (1∶50), anti-cMHC (1∶100), anti-cTnI (1∶100), anti-Connexin45 (1∶100), anti-MLC-2v (1∶100; Proteintech, Chicago, IL, USA), and anti-cTnT (1∶100; Beijing Biosynthesis Biotechnology) specific primary antibodies. The cells were then incubated with FITC (Beijing Biosynthesis Biotechnology) and phycoerythrin/PE (Proteintech) conjugated secondary antibodies for 1 h at 4°C. Finally, cells were washed with PBS and resuspended in 4% paraformaldehyde for flow cytometry. The negative control tubes were not incubated with primary antibody but with fluorescently-labeled second antibody. Data were analyzed by the FlowJo and DiVa package (Tree Star, Inc. Ashland, OR, U.S.A).

### Ca^2+^ Imaging

In order to confirm that the obtained cardiomyocytes possess functional characteristics such ascalcium transient, free intracellular Ca^2+^ imaging recordings were obtained from cells loaded with fluo-3 AM (5 µmol/L; Beyotime Institute of Biotechnology), a fluorescent Ca^2+^ indicator. Cells were incubated with fluo-3 AM at 37°C for 30 min, then washed with a standard Tyrode's buffer and incubated with DMEM at 37°C for 10 min. Video images were made by a digital camera (Canon G10, Canon, Tokyo, Japan) through a fluorescent microscope with a Nikon TE 2000 microscope (Nikon, Tokyo, Japan).

### Western blot analyses

In order to see the involvement of the ROCK signaling pathway and related other signaling pathways in the differentiation process, proteins were extracted from contracting cells with the RIPA buffer (Pierce, Rockford, IL, USA) supplemented with a protease inhibitor (Halt Protease Inhibitor Cocktail Kit, Pierce). Equivalent amounts of protein were subjected to electrophoresis, then electro-transferred onto polyvinylidene difluoride (PVDF) membranes. The membranes were blocked with 5% skim milk in a TBST (Tris Buffered Saline and 0.1% Tween 20) solution for 2 h at room temperature, then incubated at 4°C overnight with primary antibodies, including anti-JNK (1∶200, Beyotime Institute of Biotechnology), anti-phospho-JNK (1∶200, Beyotime Institute of Biotechnology), anti-Akt (1∶300, GenScript, Nanjing, China) or anti-phospho-Akt (Ser473) (1∶300, GenScript). The anti-GAPDH (1∶1000, Santa Cruz Biotechnology) or anti-β-actin (1∶1000, Santa Cruz Biotechnology) were used as an internal control. The membranes were then incubated with HRP-conjugated rabbit anti-goat and goat anti-mouse secondary antibodies (1∶2000, Santa Cruz Biotechnology) for 1 h at room temperature. All antibodies were used according to the manufacturers' recommendations. Signals were detected using a chemiluminescent ECL western blot detection system (Millipore Corp., Bedford, MA, U.S.A). The Quantity One 4.6.3 imaging software was used for densitometric analyses of the expressed protein bands.

### Statistical analyses

All experiments were performed at least three times. Main and interactive effects were analyzed by one-way ANOVAs using SPSS version 16.0 software (SPSS, Inc., Chicago, IL, U.S.A). Significance was defined as *p*<0.05.

## Results

### Differentiation of rat ADSCs into cardiomyocyte cells

**Figure 1:**
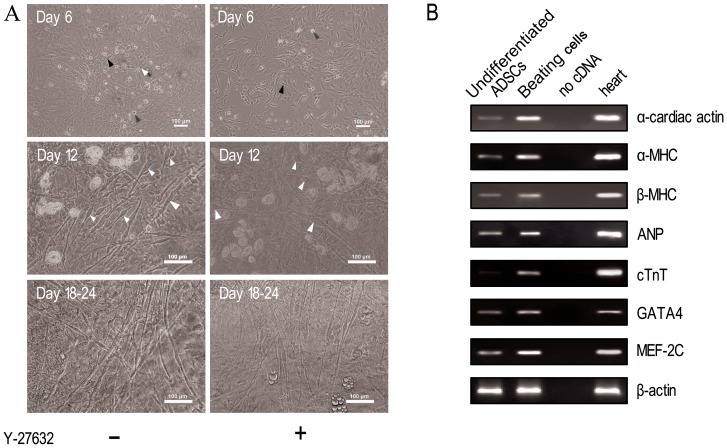
Morphology and expression of cardiac markers before and after differentiation of ADSCs into cardiomyocytes. (A) Morphological change of ADSCs into cardiomyocytes in the absence (left panels) or presence (right panels) of ROCK-specific inhibitor Y-27632. After 6 days of culture, spindle shaped cells (black arrow), small round cells (gray arrow) and small tube cells (white arrow) appeared in the top panel, with no beating. After 12 days, single contractive cells (white arrow) were shown in the center panel; After 18dyas, myotube-like structure formed a cohesive network in the bottom panels and they began to contract synchronously. Scale bars  = 100 µm. (B) Expression of cardiac genes was determined by RT-PCR before (lane 1 for undifferentiated ADSCs) and after (lane 2 for beating cells) differentiation of ADSCs into cardiomyocytes. Rat heart tissue was used as a positive control (lane 4), with no cDNA as a negative control (lane 3). Expression of α-cardiac actin, α-MHC, β-MHC, ANP, cTnT, GATA4 and MEF-2C mRNA was higher in ADSCs-derived beating cells than in undifferentiated ADSCs.

To induce the differentiation of ADSCs into beating cardiomyocytes, isolated stromal vascular fraction (SVF) cells were directly plated in the semisolid methylcellulose medium MethoCult GF M3534. We found in the absence or presence of Y-27632 did not alter the time course of ADSCs intocardiomyocytes differentiation. On the whole, after approximately 6 days of culturing, various cell morphologies began to emerge, mainly outstretched and spindle shapedcells (Fig. 1A top panel, black arrow, day6), some small round cells (Fig. 1A top panel, gray arrow, day6) and a few small tube cells (Fig. 1A top panel, white arrow, day6). Some adipocytes with lipid droplets were also observed (data not shown) at day 6. From day 9, some round, spindle-shaped and rod shape cells withsingle or multiple nuclei, began to contract rhythmically and separately (Fig. 1A center panel, white arrow, S1 Video, day 12). Within a few more days, myotube-like structure in the cells was identified, and more cells showed intense contraction, spontaneously and rhythmically (S2 Video, day 16). After 18 to 24 days, myotube-like structure formed a cohesive network (Fig. 1A bottom left, day 18) and began to contract synchronously (S3 Video, day 18). Approximately 200round cells with myotube-like structure were present in a 35 mm plate, of which nearly 72% contracted spontaneously and continued contracting for approximately several days to 1–2 weeks in the methylcellulose medium.

**Figure 2:**
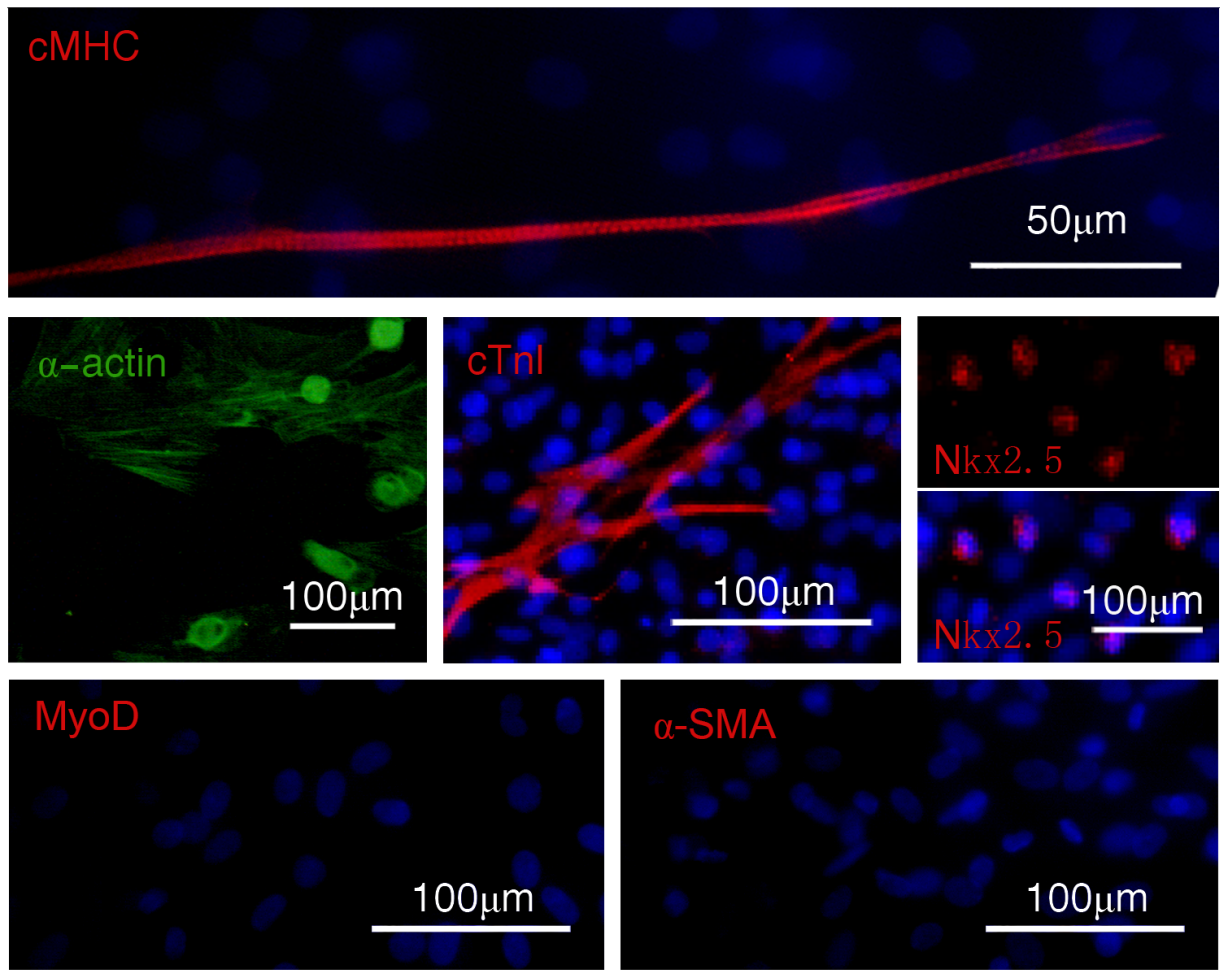
Immunofluorescence staining of contracting clone for cardiac- and muscle-proteins. Beating cells were with anti-cMHC (red), anti-α-actin (green), anti-cTnI (red) and anti-Nkx2.5 (red) antibodies. No specific staining was obtained with anti-MyoD (red) and anti-α-SMA (red) antibodies. Nuclei (blue) were stained with Hoechst 33342.

**Figure 3:**
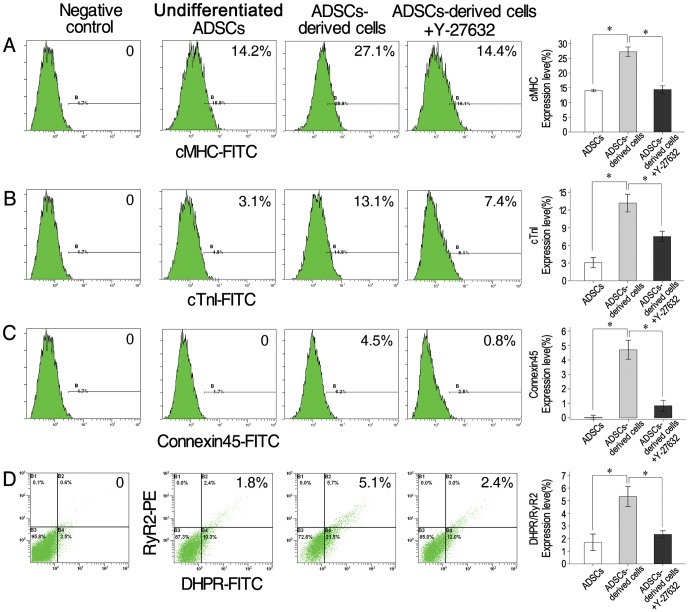
Differential protein expression by undifferentiated ADSCs and ADSCs-derived cells in the absence or presence of Y-27632. Expression of cMHC (A), cTnI (B), Connexin 45 (C) and RyR2/DHPR (D) in the ADSCs (day 3) and ADSCs-derived cells (day 16, in the absence or presence of Y-27632) by flow cytometer analyses. Right column figures represent results from 3 independent experiments. * *P*<0.05.

To confirm that the beating cells were cardiomyocytes, expression of several cardiac markersas well as a few others was examined both in undifferentiated ADSCs (adherent cells in SVF after 24 h of culture, Fig. 1B lane 1) and beating cells (cells after 16 days of culture, Fig. 1B lane 2). The results showed that cardiac mRNAs for transcription factors *GATA4* and *MEF-2C* and constitutive proteins *α-* and *β-MHC*, *cardiac α-actin*, *ANP* and *cTnT* were expressed more in ADSCs-derived beating cells than in undifferentiated ADSCs (Fig. 1B). In addition, several cardiac proteins (Nkx2.5, cTnI, cMHC, α-actin) and non-cardiac muscle proteins (MyoD for skeletal muscle and α-SMA for smooth muscle) were screened by immunofluorescence in the beating cells. Positive staining for Nkx2.5, cTnI, cMHC and α-actin was observed (Fig. 2). On the contrary, staining for MyoD and α-SMA was negative (Fig. 2), indicating these cells is not skeletal muscle cells or smooth muscle cells. To further confirm that the beating cells were cardiomyocytes, expression of cardiac proteins was also detected by flow cytometry. At day 16, the intensities of fluorescence were 27.7% (1.9-fold more than that undifferentiated ADSCs) for cMHC+, 13.2% (4.3-fold more than that undifferentiated ADSCs) for cTnI+ and 4.7% (undifferentiated ADSCs is not expression) for Connexin45+, respectively (Fig. 3A–C). Cumulatively, these results indicate that the beating cells were cardiomyocytes and they were not skeletal muscle or smooth muscle cells.

To determine whether ADSCs-derived cells possessed functional characteristics of cardiomyocytes, cardiac-specific excitation-contraction coupling (E-C coupling) dependent dihydropyridine receptor(DHPR)/L-type Voltage-dependent Ca^2+^ channels and ryanodine receptor 2(RyR2) were studied. At day 16, the intensities of fluorescence were 5.3% (3.1-fold more than that undifferentiated ADSCs) for DHPR+RyR2+by flow cytometry (Fig. 3D), indicating existence of open E-C coupling. Oscillations of intracellular Ca^2+^ measured by fluo-3 AM fluorescence intensity in spontaneous contraction cells were also observed (S4 Video).

### Involvement of ROCK in the change of cytoskeleton and the differentiation process of ADSCs-derived cardiomyocytes

**Figure 4:**
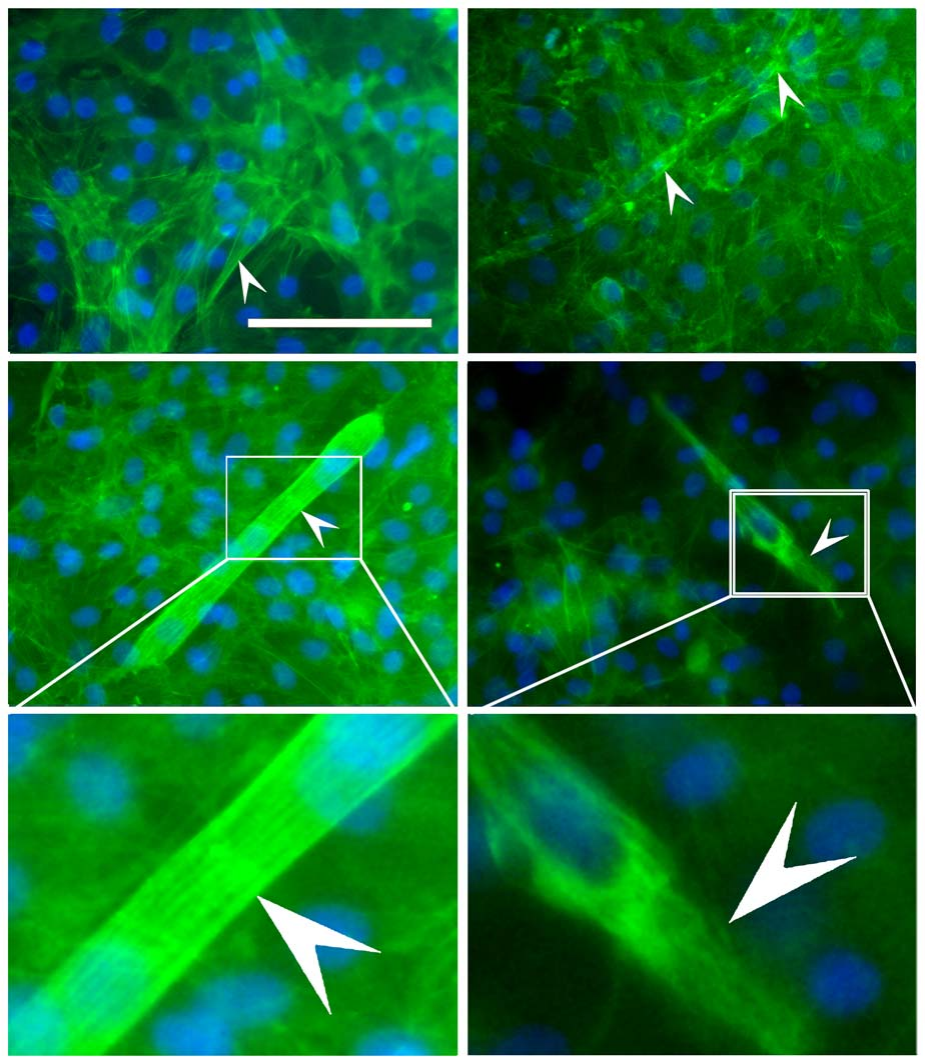
Morphological change of actin cytoskeleton during the differentiation of ADSCs. ADSCs and ADSCs-derived cardiomyocytes were stained with phalloidin for F-actin (green). The white arrows point to stained stress fibers. After 3 days of culture in the absence of Y-27632 (top left panel), stress fibers in cells weres parse. After 7 days of culture in the absence of Y-27632 (top right panel), stress fibersshifted from sparse to dense distribution. The long stress fibers fully occupied the cytoplasm of contractive cells after 16 days of culture in the absence of Y-27632 (middle left and bottom left panels). The contractive cells in the presence of Y-27632 showed much less compact stress fibers in the cytoplasm, in comparison with un-treated cells (after 16 days of culture, middle rightand bottom right panels). The white arrow indicates stress fibers. Nuclei (blue) were stained with Hoechst 33342, Scale bar  = 100 µm.

Since the differentiation process involves change of cell shape, rearrangement of actin cytoskeleton was studied by phalloidin staining during differentiation of ADSCs into cardiomyocytes. Intracellular structure of filamentous actin shifted from sparse (Fig. 4 top left panel, white arrow) in undifferentiated ADSCs to dense distribution (Fig. 4 top right panel, white arrow) in the differentiating ADSCs. In beating cardiomyocytes, long stress fibers fully occupied the cytoplasm (Fig. 4 middle left and bottom left panels, white arrow), responsible for strong contraction of ADSCs-derived cardiomyocytes. All in all, in addition to the morphological canges, density of stress fiber is increased with the cardiomyocytes differentiation process.

**Figure 5:**
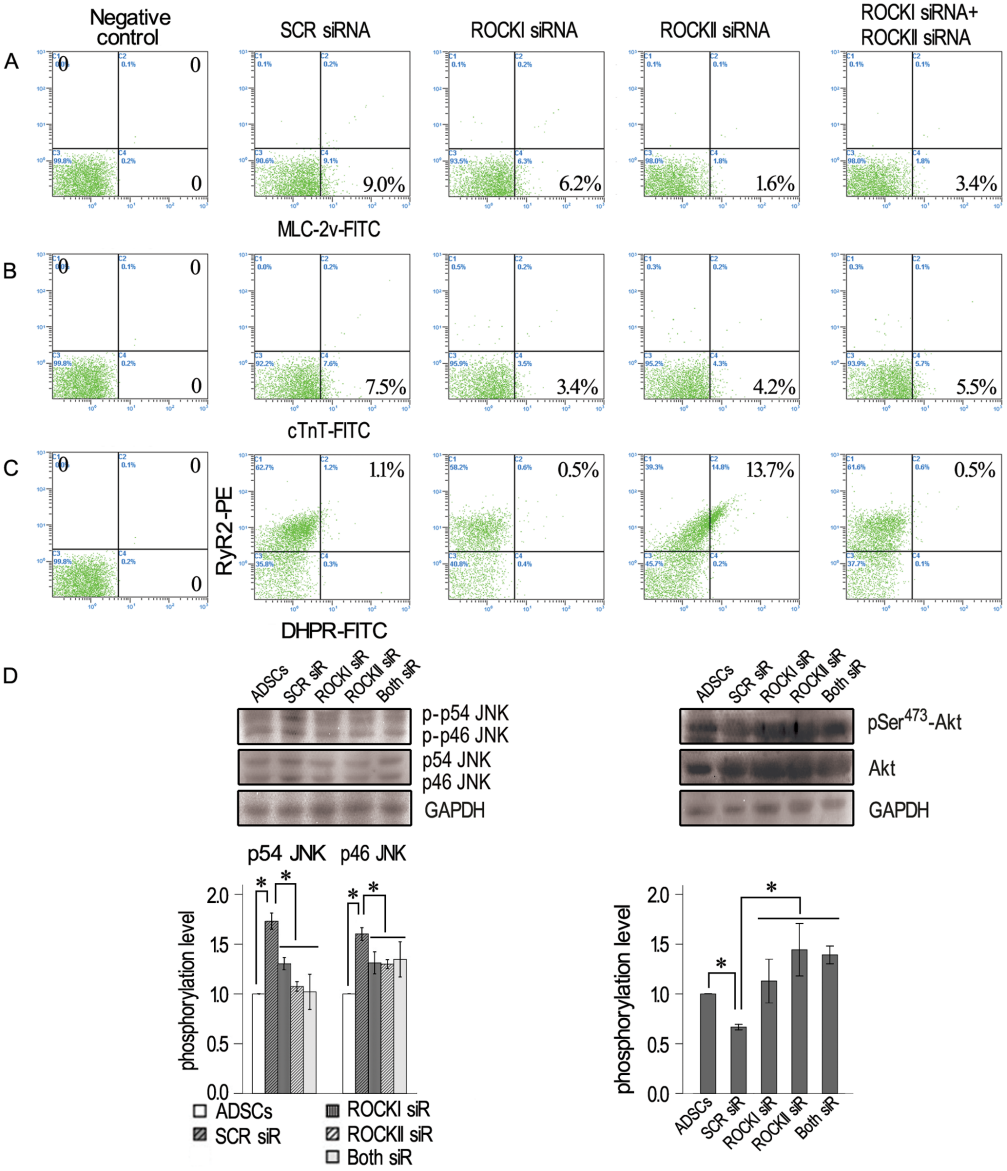
Expression of cardiac proteins, excitation-contraction coupling proteins and signaling proteins in beating cardiomyocytes. Expression of MLC-2v(A), cTnT(B) and RyR2/DHPR (C) in the SCR siRNA and ROCK siRNA groups by flow cytometer analyses. (D) Change of JNK and PI3K/Akt from Western Blot analyses. Lane 1 represents undifferentiated ADSCs (day 3), while lanes 2 to 5represent SCR siRNA, ROCKI siRNA, ROCKII siRNA and Both siRNA groups, respectively (day 16). Phosphorylation level  =  phosphorylation/total. * *P*<0.05.

It is well known that cytoskeleton change is regulated by ROCKs. To determine the role of ROCKs in the differentiation process, SVF cells on the MethoCult GF M3534 were treated with 10 µM of ROCK inhibitor Y-27632 for 16 days. The treated cells showed much less compact stress fibers in the cells (Fig. 4 middle right and bottom right panels, white arrow), in comparison with un-treated cells (Fig. 4 middle left and bottom left panels, white arrow). More importantly, when cardiomyocyte-like cells in the control group without Y-27632 began to beat, compared number of contracting cells in the two groups. We found that addition of Y-27632 did not delay the formation of contractive cells, but the number of contractive cells were significantly reduced by approximately 31%. The results obtained with the ROCK inhibitor were confirmed by using siRNA. Two isoforms of ROCKs were inhibited by siRNA transfection. When expression of cardiac proteins MLC-2v, cTnT, DHPR and RyR2 was detected by flow cytometryon day 16, the numbers of cells expressing MLC-2v and cTnT in the ROCKI siRNA, ROCKII siRNA or both ROCKI siRNA + ROCKII siRNA groups decreased in comparison with the SCR siRNA group at the same time point (Fig. 5A–C). The ROCKII siRNA increased the numbers of cells expressing DHPR+RyR2+, while ROCKI siRNA or the ROCKI siRNA + ROCKII siRNA did not have any effect (Fig. 5A–C). These results proved that inhibition of ROCKI and ROCKII reduced the expression cardiac proteins and the formation of cardiomyocytes.

### ROCKs interact with other signaling pathways during the differentiation process

**Figure 6:**
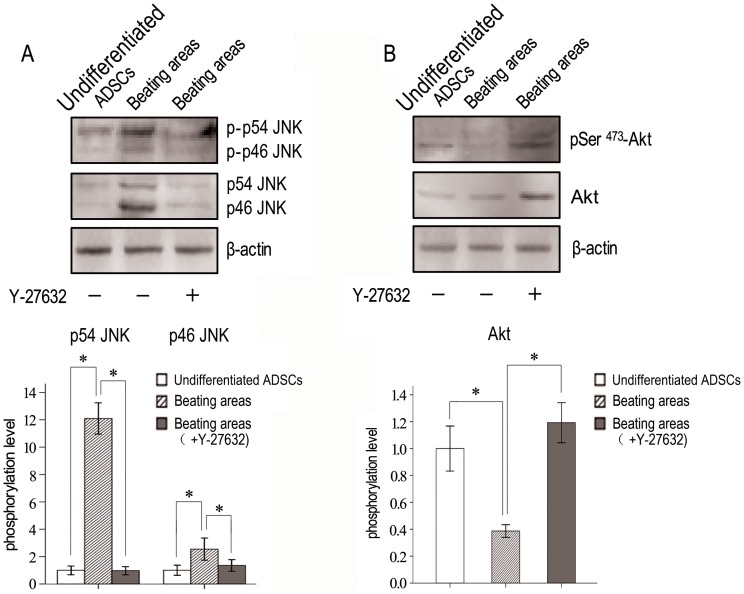
Changes of ROCK related signaling molecules before or after the differentiation of ADSCs into cardiomyocytes. (A) Phosphorylation levels of JNK. (**B**) Phosphorylation level of Akt. Lane 1 represents undifferentiated ADSCs (day 3). Lanes 2 and 3 representbeating cells (day 16) in the absence or presence of Y-27632, respectively. Phosphorylation level  =  phosphorylation/total. * *P*<0.05.

Mitogen-activated protein kinases (MAPKs) have been implicated as regulators of differentiation. Similarly, thePI3K/Akt pathway appears to promote growth arrest and differentiation. In order to see whether there is cross-talk between ROCKs and other pathways involved in the differentiation, the activities of one MAPK pathway member, Jun N-terminal kinases (JNKs), and one PI3K/Akt pathway member, Akt, were studied before and after differentiation. The total protein was collected from the contractive area (day 16) and undifferentiated ADSCs (day 3). As shown in Fig. 6, compared with that before differentiation, the level of JNK phosphorylations (JNK1, 12.1-fold increase, *p<0.05*; JNK2/3, 2.5-fold increase, *p<0.05*) in the contractive area was improved significantly (Fig. 6A), but the level of Akt phosphorylations which is one component of PI3K/Akt signal pathway relating to ROCK was reduced obviously (61.3% decrease, *p<0.05*) (Fig. 6B). Y-27632 significantly inhibited both total JNK and JNK phosphorylation (91.9% decrease for p54 JNK, *p*<0.05; 46.8% decrease for p46 JNK, *p*<0.05) (Fig. 6A). At the same time, Y-27632–induced Akt phosphorylation (3.1-fold increase, *p*<0.05) was increased significantly (Fig. 6B). Similar results were obtained with ROCK siRNAs. Akt phosphorylation was significantly increased, both total JNK and JNK phosphorylation was decreased after ROCK siRNA treatments (Fig. 5D).

## Discussion

Treatment of damaged cardiac muscle by cell transplantation is a promising strategy to regain cardiac functions. ADSCs are abundant and easy to get and proliferate fast in vitro. They can be used for autologous transplantation and the damage to the donor site is not serious. Thus, transplantation of ADSCs or ADSC derived cardiomyocytes to MI patients is receiving increasing attention. There are several ways to obtain cardiomyocytes from adipose tissues: direct implantation, culturing cells on semi-solid culture, chemical treatments and dedifferentiation of fat (DFAT) cells. Except DFAT cells, all other methods have very low differentiation ratesand need stringent differentiation condition. DFAT cells are relatively pure and relatively efficient to be differentiated into the beating cardiomyocyte (about 10%) [21]. However, the biological safety of their implantation requires more long term animal or preclinical studies, considering the occurrence of dedifferentiated liposarcoma in nature [21]. Isolation of cell types from ADSCs which can differentiate into beating cardiomyocytes efficiently and optimization of differentiation condition continue to be major areas for future research.

In this study, we show, for the first time, that spontaneous contractile cardiomyocytes could be derived from rat ADSCs after culturing in the MethoCult GF M3534. The following evidence supports the notion that the differentiated cells were cardiomyocytes. First, MyoD and α-SMA were not detected at different phases during cardiomyocyte differentiation and indicated that the contractive cells were not skeletal muscle and smooth muscle. Second, several well recognized cardiomyocyte markers including Nkx2.5, ANP, cTnI, MLC-2v and cTnT were detected. Furthermore, detection of DHPR and RyR2 (RyR2 is a marker of cardiac muscle, while RyR1 is a marker of skeletal muscle) was positive and imaging of calcium transient was observed. Similarto results reported by others [6], [9], [22], we observed that some spontaneously beating cardiac myocytes are myotube-like. While they appeared like skeletal myocytes, these cells expressed cardiac markers including high expression of Nkx2.5. It is worth of pointing out that skeletal myoblasts and myocytes also express Nkx2.5. However, expression levels of Nkx2.5 decrease as C2C12 myoblasts elongate and fuse to form myotubes [23]. In addition, expression of human Nkx2.5 in C2C12 myoblasts inhibits myocyte differentiation and myotube formation and expression ofNkx2.5 in terminally differentiated C2C12 myotubes results in breakdown into smaller myotubes [23]. Such phenomena were not observed in this study, providing additional support to the notion that myotube-like cells in this study are cardiac myocytes.

Compared with mouse ADSCs-derived functional cardiomyocytes [6], appearance of rat ADSCs-derived functional cardiomyocytes was earlier than that of mice (9–14 d for rats and 11–14 d for mice), while the beating period of cardiomyocytes in rats was shorter than that of mice (about several days to 2 weeks for rats and a few months for mice). We suppose that rat ADSCs might be more sensitive to certain factors within MethoCult GF M3534, which promoted adipogenic differentiation of preadipocytes within rat ADSCs and accelerated the formation of mature adipocytes. Consequently, cardiomyogenesis of rat ADSCs was earlier than that of mouse ADSCs. Lamounier-Zepter et al. [24] reported that fatty acid binding proteins secreted by mature adipocytes suppressed contraction of cardiomyocytes through attenuating intracellular Ca^2+^ levels. Therefore, the adipogenesisin our ADSCs culture might be responsible for the shorter pulsatile period of cardiomyocyte in rats. The main pitfall for this study is no in vivo confirmation of the functionality of beating cardiomyocytes. However, many reports have showed that transplantation of ADSCs or ADSCs derived cardiomyocytes could improve cardiac functions after both acute and chronic infarction [16], [25]–[27]. Bai et al. [28] also showed that engrafted ADSCs took a cardiomyogenic and vascular cell differentiation pathway without evidence of cell fusion. In addition, the injected cells promoted angiogenesis and reduced the apoptosis rate in cardiomyocytes at the border zone [28]. Nevertheless, various animal models need to be fine-tuned in terms of induction of ischemic myocardium, numbers of cells injected, route of cell delivery and enhancement of retention and ways for survival of transplanted cells.

Another major finding from this study was involvement of the ROCK pathway in the differentiation process of ADSCs into cardiomyocytes. A further understanding of the biomechanical and biochemical pathways involved in signaling transduction will hopefully provide new insights for improvement of stem-cell based therapies. Cell shape is structurally regulated primarily by actin microfilaments and microtubules [18], [29]. ROCKs are one of the critical downstream effectors of Rho GTPases and regulate diverse cellular functions including cell migration, actin cytoskeletal organization and stress fiber contraction. In the present study, we clearly demonstrated that the differentiation of ADSCs into cardiomyocytes involved marked change of stress fiber. Inhibition of ROCKs loosened the stress fiber in cytoplasm, and reduced the number of cells expressing cardiomyocyte markers and contracting. These findings suggest that inhibition of ROCKs weakened the differentiation of ADSCs into cardiomyocytes. ROCK proteins phosphorylate an abundant array of downstream targets, which modify filamentous-actin (F-actin) ultra-structural assemblies that are important for the regulation of cell contractility, motility and morphology [30]. In this study, activation of JNK and inhibition of Akt were associated with the differentiation of ADSCs into beating cardiomyocytes. The involvement of Akt in cardiomyocyte differentiation was clearly demonstrated by Ishida et al. [31], when they studied LEOPARD syndrome, a rare autosomal dominant disease caused by a mutation in the non-receptor type 11 gene (*PTPN11*). They generated mutant P19CL6 cell lines and showed that the mutation attenuated terminal differentiation of cardiomyocytes via hyperphosphorylation of Akt [31]. The results from Ishida et al. support our observation that ROCK inhibitor Y-27632 or ROCK siRNA–induced Akt phosphorylation was correlated with reduced differentiation of ADSCs into cardiomyocytes. Mitogen-activated protein kinases (MAPKs) are signaling components that are important in converting extracellular stimuli into a wide range of cellular responses. One of the MAPKs, JNK, was investigated in this study. The role of JNK in the differentiation to cardiomyocytes is less clear. The fact that 30% of wide type mouse embryonic stem cells and 60% of MAP2K7-deficient mouse embryonic stem cells developed rhythmic contractile cardiomyocytes suggests the inhibitory effect of JNK on the differentiation, since ablation of the upstream MAP kinase kinase (MAP2K7) in embryonic stem cells prevented JNK activation [32]. On the other hand, our result on JNK is in agreement with Rai et al. [33], who observed that blocking the initial noncanonical JNK/AP-1 signaling with SP60125 aborted cardiovascular differentiation and promoted hematopoiesis from mouse embryonic stem cells.

## Conclusions

Based on our results, rat ADSCs could spontaneously differentiate into cardiomyocytes in vitro. This finding could expedite the research of cardiovascular diseases in a rat model. Furthermore, ROCKs play an important role in the differentiation of ADSCs into beating cardiomyocytes in conjunction of the PI3K/Akt pathway and the JNK pathway.

## Supporting Information

S1 Video **At day 9–14, some cells began to contract rhythmically separately.**
(MPG)

S2 Video **At day 16, more and more cells showed intensively, spontaneously and rhythmically contraction.**
(MPG)

S3 Video **Myotube-like structure gave rise to a cohesive network.**
(MPG)

S4 Video **Oscillations in fluo-3 AM fluorescence intensity in these cells were observed.**
(MPG)
